# Rheumatic Diseases: New Progress in Clinical Research and Pathogenesis

**DOI:** 10.3390/medicina59091581

**Published:** 2023-08-31

**Authors:** Malcolm Koo, Ming-Chi Lu

**Affiliations:** 1 Department of Nursing, Tzu Chi University of Science and Technology, Hualien 970302, Taiwan; m.koo@utoronto.ca; 2 Dalla Lana School of Public Health, University of Toronto, Toronto, ON M5T 3M7, Canada; 3 Division of Allergy, Immunology and Rheumatology, Dalin Tzu Chi Hospital, Buddhist Tzu Chi Medical Foundation, Dalin, Chiayi 622401, Taiwan; 4 School of Medicine, Tzu Chi University, Hualien 970374, Taiwan

Rheumatic diseases encompass a group of disorders that primarily target the musculoskeletal system, including joints, bones, muscles, and connective tissue. Common rheumatic diseases, such as rheumatoid arthritis (RA), systemic lupus erythematosus (SLE), primary Sjögren’s syndrome, and ankylosing spondylitis (AS), are chronic diseases that result in the dysregulation of the immune system [1]. These diseases often involve multiple organ systems, leading to significant morbidity and mortality among those diagnosed with rheumatic diseases [2,3]. Managing these diseases presents challenges not only for physicians but also for rheumatologists. The COVID-19 pandemic further complicated the management of patients with rheumatic diseases [4].

This Special Issue of *Medicina*, entitled “Rheumatic Diseases: New Progress in Clinical Research and Pathogenesis”, features ten articles focusing on applying standard Western medications, Chinese medicine, and complementary therapies to patients with rheumatic diseases, increasing incidence of morbidity of these diseases, and changing mortality rates during the COVID-19 pandemic period, representing critical issues within the field.

Téllez Arévalo et al. [5] have provided us with a comprehensive review concerning the mechanism of action, efficacy, safety, and, most importantly, monitoring parameters of important synthetic drugs used in the treatment of SLE. SLE serves as a prototype for systemic autoimmune diseases. This article provided a valuable resource for physicians seeking to become familiar with SLE treatment. In addition to traditional immunosuppressants, biologic agents such as rituximab and belimumab are recommended for patients with SLE who demonstrate inadequate responses to standard therapies [6]. Capdevila et al. examined the characteristics and the predictive factors of rituximab and belimumab use in real-world practice among patients with SLE [7]. They found that rituximab was primarily used for conditions like hemolytic anemia or thrombocytopenia, lupus nephritis, and neuropsychiatric lupus, whereas belimumab was mainly used for arthritis. Currently, more biologic agents, such as anifrolumab, are available [8]. The integration of biologics could enhance the clinical outcomes for those with SLE.

Besides Western medicine, Chinese medicine and complementary therapies have been extensively investigated for their potential use in treating rheumatic diseases [9,10]. Liao et al. demonstrated that patients with RA using Chinese medicine could reduce the risk of developing Sjögren’s Syndrome, a common extra-articular manifestation of RA [11]. Regarding complementary therapy usage, our previous study found that more than 85% of Taiwanese patients with SLE used complementary therapies on a regular basis [12]. We found that different clinical manifestations of SLE were associated with the use of specific complementary therapies. For example, Raynaud’s phenomenon was significantly associated with fitness walking or strolling, and fish oil supplements. In contrast, photosensitivity was associated with probiotics and white renal involvement, with both probiotics and visits to the Chinese medicine department in hospitals [13]. These associations might guide future research toward possible efficacious therapies for SLE.

Mahmoud et al. evaluated Siwan sand therapy, a traditional treatment in Egypt for alleviating joint pain. They found that five days of Siwan traditional therapy could reduce inflammation, improve the lipid profile, and enhance the quality of life in patients with RA compared to those receiving only three days of this therapy [14]. Although a systematic review indicated that limited evidence supports the therapeutic effect of hot sand baths on symptoms and functionality in patients with rheumatic diseases [15], the potential impact of hot sand baths on cardiovascular diseases and quality of life in patients with RA merits further study.

Cardiovascular disease is an important cause of death in patients with rheumatic diseases [16]. Bedeković et al. reviewed the effect of RA on the cardiovascular system [17]. They concluded that chronic inflammation and some medication for treating RA could accelerate atherosclerosis, and that maintaining long-term remission using novel therapeutic agents in RA may prevent cardiovascular events. Chronic lung disease is another leading cause of morbidity and mortality in patients with rheumatic diseases. Recently, the association between RA and the airway has been reported [18]. It is known that anti-melanoma differentiation-associated gene 5 (MDA5) antibodies (Abs) are linked with amyopathic dermatomyositis developing into rapidly progressive interstitial lung disease (ILD). Higher levels of anti-MDA5 Abs were found in RA patients with airway disease compared to those without [19]. Investigating the role of anti-MDA5 Abs in the pathogenesis of lung diseases in patients with rheumatic conditions can be a fruitful avenue of research.

Bones and joints are often the direct targets of rheumatic diseases, and it is unsurprising that patients with rheumatic diseases are seriously affected by joint deformity and bone damage. These individuals may experience a higher likelihood of undergoing orthopedic surgeries. Chen et al. revealed that patients with RA had an increased risk of receiving lumbar spine surgery, with older age and concurrent osteoporosis as risk factors [20]. Our group has been investigating this issue for years, summarizing the risks associated with four common orthopedic surgeries, including total knee replacement, total hip replacement, cervical spine surgery, and lumbar spine surgery, in prevalent rheumatic diseases including RA, SLE, AS, and psoriasis [21]. Given that joint deformity and bone damage in rheumatic patients take time to develop, we hope that the implementation of biologic agents and a treat-to-target strategy could minimize the need for orthopedic surgeries.

The recent COVID-19 pandemic has significantly impacted global health, including those with rheumatic diseases who have impaired immunity. Therefore, COVID-19 infections undoubtedly affect their clinical outcomes. Dadonienė et al. found that patients with rheumatic diseases had a lower mortality rate than expected in Lithuania [22]. Stringent lockdown measures, social distancing, and early vaccination can reduce the incidence of influenza and other infectious respiratory diseases. Though most restrictions have lifted with the end of the COVID-19 pandemic, continuing personal protective behaviors like handwashing, wearing masks, and regular vaccination is vital to prevent infections that could be fatal for those with weakened immunity.

The articles published in this Special Issue offer fresh perspectives on the clinical care of patients with rheumatic diseases. Rheumatologists can benefit from these insights, leveraging them to reduce patient morbidity and mortality.
